# Pediatric Elbow Fractures in a Major Trauma Center in Iran

**DOI:** 10.5812/atr.8098

**Published:** 2013-02-01

**Authors:** Akbar Behdad, Samin Behdad, Mehrdad Hosseinpour

**Affiliations:** 1 Department of Surgery, Faculty of Medicine, Isfahan University of Medical Sciences, Isfahan, IR Iran; 2 Trauma Research Center, Kashan University of Medical Sciences, Kashan, IR Iran

**Keywords:** Elbow, Bone Fractures, Epidemiology, Pediatrics

## Abstract

**Background:**

Elbow fractures are one of the most common traumatic fractures in the pediatric population. Since severe complications may occur, appropriate diagnosis and treatment are imperative when dealing with this type of fracture.

**Objectives:**

The aim of this study was to evaluate the epidemiology of elbow fracture in children admitted in Alzahra hospital, Isfahan, IR Iran over a one year period.

**Patients and Methods:**

During a one year period, a prospective study was conducted on 300 patients under the age of 16 who had sustained elbow fractures. Data included age, gender, mechanism of trauma, type of elbow fractures, complications, and outcomes.

**Results:**

The mean age of the patients was 8.1 ± 2.31 years old. Boys were injured 2.6 times more often than the girls. Falling was the major cause of pediatric elbow fractures (86%). Supra condylar were the most common type of fracture. There was a significant association between gender and type of injury (P < 0.01).

**Conclusions:**

Supracondylar fracture is the most common fracture type resulting from 4 - 8 year old boys’ falls. Our findings indicate the critical nature of appropriate treatment in order to prevent severe complications.

## 1. Background

Elbow fractures are among the most common traumatic problem in the pediatric population and consist of approximately 15% of the all pediatric fractures (1). In some studies, the cause of 85% of orthopedic surgeries is elbow fracture (2, 3). These fractures consist of supracondylar, lateral condyle, neck of radius, medial epicondyle, olecranon, head of radius and intercondylar fractures. Supracondylar fracture is the most common fracture in children under seventeen years of age. Fracture of any extremity is rarely life-threatening, but it may cause major morbidity, inability to work, increased disability-adjusted life year (DALY) and severe psychological distress (4). Due to serious complications, timely diagnosis and treatment are considered as very important issue in this type of fracture. The unique anatomy and the intimate location of neurovascular structures often result in a spectrum of injuries with associated complications (Cobitus varus, neurovascular injuries, and Volkman ischemic syndrome) and potential long-term disabilities (5, 6). The incidence of traumatic and iatrogenic nerve injuries with this type of fracture have been recorded as 12 – 20% and 2 – 6%, respectively (7).

## 2. Objectives

The former Iranian studies conducted by Gorji (8) and Khaji (9) for the evaluation of epidemiology and mechanism of elbow fractures in children have limitations, and accurate data about the epidemiology of pediatric elbow fractures in Iran can only be achieved by the accumulation of data from all parts of the country. We conducted this study to assess the permutations of these injuries in children in one of the major trauma centers in Isfahan.

## 3. Patients and Methods

A descriptive case series study was carried out on 300 pediatric patients (all under sixteen years old) who were admitted to Alzahra educational hospital, Isfahan, Iran, from April 2011 to March 2012. Data about the patients and characteristics of their injuries were collected using patients’ records. Radiographic studies (anterior–posterior and lateral view) were performed in order to confirm the elbow fracture and following verification from the specialist, patients were admitted for a final evaluation. Etiology of injury, demographic data (age, gender), type of fractures and left or right arm dominancy were collected. Collected data were classified and reported in distribution of frequency tables. For evaluation, the relationship between type of fracture, age, gender and injured hand, Chi –Square or Exact Fissure’s test was utilized. Results were considered significant at P ≤ 0.05.

## 4. Results

Of the 300 patients admitted to hospital due to an accident, 217 cases (72.3%) were male and 83 (27.7%) female. The mean age of patients was 8.1 ± 2.31 years. Table 1 illustrated the frequency of distribution according to age. According to this table, the age group of the patients was 4 - 8 years. Table 2 illustrated the distribution of the type of fracture. According to this table, supracondylar fracture is the most common type in patients under sixteen years. Accidental falls accounted for 86% of supracondylar fractures. Other etiologies were traffic accidents (13.2%) and violence (8%). One hundred and seventy six patients (58.7%) have left elbow fracture and 124 of them (41.3%) suffered a right elbow fracture. Table 3 illustrated the frequency distribution of the type of fractures according to the age groups. As stated in this table, supracondylar fractures are more common in 4-8 years old children (P = 0.05). Furthermore, Table 4 illustrated the frequency distribution of the type of injury according to gender. Elbow fractures were more common in the male gender (P = 0.01). There were no significant differences between the dominancy of arm and fracture types (Table 5). The types of supracondylar fractures were not mentioned in patients’ records.

**Table 1.** Distribution of Elbow Fracture According to Age Group

Age Group, y	No. (%)
**0 - 4**	58 (19.3)
**4 - 8**	113 (37.7)
**8 - 12**	76 (25.3)
**12 - 16**	53 (17.7)

**Table 2.** Distribution of Elbow Fracture According to Type of Fracture

Type of Fracture	No. (%)
**Supra condylar**	174 (58.0)
**Lateral condyl**	28 (9.3)
**Neck of radius**	22 (7.3)
**Medial epicondyl**	13 (4.3)
**Olecranone**	16 (5.3)
**Head of radius**	32 (10.7)
**Inter condylar**	9 (3.0)
**Mix**	6 (2.0)

**Table 3.** Frequency of Type of Elbow Fracture According to Age Group

Age Group, y	Supra Condylar, No.	Head of Radius, No.	Lateral Condyle, No.	Neck of Radius, No.	Olecranon, No.
**0 - 4**	35	6	4	4	4
**4 - 8**	70	9	15	7	5
**8 - 12**	47	11	6	7	2
**12 - 16**	22	6	3	4	5

**Table 4.** Frequency of Type of Fracture According to Gender

Type of Fracture	Male, No.	Female, No.
**Supra condylar**	125	49
**Head of radius**	17	15
**Neck of radius**	16	6
**Lateral condyle**	21	7
**Olecranon**	13	3

**Table 5.** Frequency of Elbow Fracture According to Side of Injury

Type of Fracture	Right, No.	Left, No.
**Supra condylar**	68	106
**Head of radius**	13	19
**Lateral condyle**	13	15
**Neck of radius**	9	13
**Olecranon**	6	10

## 5. Discussion

In this study, supracondylar fracture was presented in 58% of all cases and was the most common cause of elbow fracture in patients under 16 years of age, a finding that was similar to the Jessica study (10). The injury was on the outstretched arm which resulted in hyperextension of the elbow and was caused by falling from a height. Falls and traffic crashes were the main causes of injuries, with the percentages of 86% and 13.2% that in Khaji study were 57.3% and 37.1%, respectively (9). Association between age groups and supracondylar fracture was repeated in other studies. The incidence of this type of fracture peaks between 2 - 7 years (11). This peak is thought to be associated with the fact that the capsule and ligaments supporting the elbow have been revealed to have greater tensile strength than the bone itself, which leads to preferential fracture of the vulnerable supracondylar region when sufficient force is applied across it. Seventy-five percent of supracondylar fracture occurred in males, which correlates to the Marquis study (11). Uncontrolled and unlimited playing for boys such as cycling and playground injuries make the male gender more vulnerable to fracture (12). In our study, there was no significant relation between dominant elbow and fracture but most of them were in the left elbow as stated by Igbigbi (13). In Chai and Chakraborty studies, the non-dominant arm was more frequently affected than the dominant arm (14, 15). There is no definitive etiology for the more common left elbow fracture but the explanation is that most patients were right dominant and since the left arm is weaker than the other arm, the force of the fall caused the fracture in the weaker elbow. In the present study, fracture of the head of the radius was common following the supracondylar fracture which is the inverse of Biruk’s study. In his study, the next common type was a medial condylar fracture (16) as well as in Lipczyk’s study, the lateral humeral condyle was the second most common elbow fracture in children after supracondylar fracture of the humerus (17). Finally, a recent study showed that supracondylar fractures are the most common particularly in boys aged 4 - 8 years after falling. To avoid severe complications, an accurate diagnosis and in time treatment is crucial. The present study has the usual limitations of being a retrospective study when it comes to collection of data about the fractures and early complications.
